# Characterizing Local Dynamic Stability of Lumbar Spine Sub-regions During Repetitive Trunk Flexion-Extension Movements

**DOI:** 10.3389/fspor.2019.00048

**Published:** 2019-10-10

**Authors:** Dennis J. Larson, Yunxi Wang, Derek P. Zwambag, Stephen H. M. Brown

**Affiliations:** ^1^Spine and Muscle Biomechanics Lab, Department of Human Health and Nutritional Sciences, University of Guelph, Guelph, ON, Canada; ^2^Department of Kinesiology and Physical Education, Wilfrid Laurier University, Waterloo, ON, Canada

**Keywords:** lumbar, intervertebral, spine, local dynamic stability, lyapunov exponents

## Abstract

Using a technique of tracking intersegmental spine kinematics via skin surface markers, this study aimed to estimate local dynamic spine stability across smaller sub-regions (or segments) of the lumbar spine while also considering the impact of an external pelvic constraint during repetitive movements. Sixteen participants (10 males) performed two trials [Free Motion (FM), Pelvis Constrained (PC)] each consisting of 65 repetitive trunk flexion-extension movements to assess dynamic spine stability using maximum Lyapunov exponents (LyE). First, results indicated that LyE obtained from analysis of 30 repetitive flexion-extension movements did not differ from those obtained from analysis of greater numbers of repetitive movements, which aligns with results from a previous study for the whole lumbar spine. Next, for both males and females, and FM and PC trials, the most caudal region of the lumbar spine behaved the most dynamically stable, while upper lumbar regions behaved the most dynamically unstable. Finally, females demonstrated greater lumbar and intersegmental stability (lower LyE) during PC trials compared to FM, while males demonstrated slightly decreased lumbar and intersegmental stability (higher LyE) during PC trials compared to FM; this resulted in PC trials, but not FM trials, being different between sexes. Altogether, these data show that dynamic stability of lumbar spine sub-regions may be related to the proximity of the motion segment to rigid skeletal structures, and that consideration is needed when deciding whether to constrain the pelvis during analyses of dynamic spine stability.

## Introduction

Information regarding neuromuscular control of human movement can be obtained through the application of non-linear dynamics analyses of repetitive motion patterns. Using repetitive lumbar spine flexion-extension movements, local dynamic stability of the lumbar spine was first evaluated by Granata and England (2006). Many studies have since advanced our understanding of spine neuromuscular control by investigating how variables including movement speed (e.g., Granata and England, 2006; Graham and Brown, 2012; Asgari et al., 2015), external load (e.g., Graham and Brown, 2012; Beaudette et al., 2014), low back pain (e.g., Graham et al., 2014; Ross et al., 2015), muscle fatigue (e.g., Granata and Gottipati, 2008; Asgari et al., 2017; Larson et al., 2018), and task asymmetry (Dupeyron et al., 2013; Lee and Nussbaum, 2013) affect dynamic lumbar spine stability.

Studies investigating spine motion have most commonly affixed rigid bodies or sensors to the skin over the participant's pelvis and thorax to obtain 3-dimensional (3D) angular kinematics of the whole lumbar spine (e.g., Howarth, 2014). This has enabled the study of lumbar spine dynamic stability under a variety of task conditions; however, only inferences could be made regarding intervertebral or intersegmental spine motion. Knowing that abnormal intervertebral spine motion/stability has been considered a risk factor for low back pain and injury (Cholewicki and McGill, 1996; McGill and Cholewicki, 2001), having the ability to measure lumbar motion at a higher spatial resolution (i.e., intersegmentally) during dynamic movements will improve our knowledge and understanding of spine movement control. With recent developments in the resolution with which spine skin-surface motion is tracked (Zwambag et al., 2018), we can now estimate dynamic stability across smaller spine regions or segments and the whole lumbar spine concurrently.

Considering the complex coordinated motion of the lower limbs, pelvis, and trunk during dynamic trunk movements, understanding the neuromuscular control of the spine becomes increasingly difficult. To simplify, researchers have often constrained motion to decrease the degrees of freedom involved in the movement; however, this may limit our understanding of functional human motion (Delphinus and Sayers, 2013). In regard to dynamic spine stability, researchers have either constrained lower limb/pelvis motion to study the spine system in isolation (e.g., Granata and England, 2006; Graham et al., 2014; Ross et al., 2015; Larson et al., 2018) or have studied spine motion free of any constraints (e.g., Graham and Brown, 2012; Dupeyron et al., 2013; Lee and Nussbaum, 2013; Asgari et al., 2015), with only two known studies comparing both conditions (Granata and Gottipati, 2008; Howarth and Graham, 2015). From these latter studies, no clear consensus regarding the effects of a pelvis constraint can be made as Granata and Gottipati (2008) observed a decrease in dynamic spine stability (higher LyE), while Howarth and Graham (2015) observed an increase in dynamic spine stability (lower LyE) when using an external pelvis constraint. Therefore, more research is needed to compare free motion (FM) and pelvis constrained (PC) movements, as this may be of particular importance when higher spatial resolution measures and comparisons are made between lumbar spine sub-regions of different proximities to the pelvis. Moreover, possible differences in how males and females move their spines in both free motion and pelvic constrained conditions have not been adequately explored.

Altogether, the primary goal of the current investigation was to calculate and compare dynamic spine stability across lumbar spine sub-regions (i.e., intersegmentally). A secondary goal was to determine if a pelvic constraint affected all sub-regions of the lumbar spine similarly. We hypothesized that lower lumbar segments would behave the least dynamically stable (higher LyE) across both movement conditions (FM and PC), as lower segments are often linked to lower back pain and injury through abnormal intervertebral motion/stability (Friberg and Hirsch, 1949; Cholewicki and McGill, 1996; McGill and Cholewicki, 2001). Additionally, we hypothesized that both whole lumbar and intersegmental dynamic stability would be greater (lower LyE) when constraining the pelvis as this acts to decrease the degrees of freedom involved in the movement.

## Materials and Methods

### Participants

Sixteen participants (6 F, 10 M) with no recent history (<3 months) of low back pain/injury or any musculoskeletal disorders volunteered to participate in the study (Table 1). All participants completed a health screening questionnaire and provided informed consent prior to participating the study, which was approved by the University Research Ethics Board.

**Table 1:** Participant mean (±SD) demographics.

**Demographic**	**Female (*n* = 6)**	**Male (*n* = 10)**
Age (years)	25 ± 2.7	24 ± 3.3
Height (cm)	167 ± 9.6	179 ± 5.9
Mass (kg)	59 ± 8.5	82 ± 12.0

### Intersegmental Kinematics

**Figure 1:**
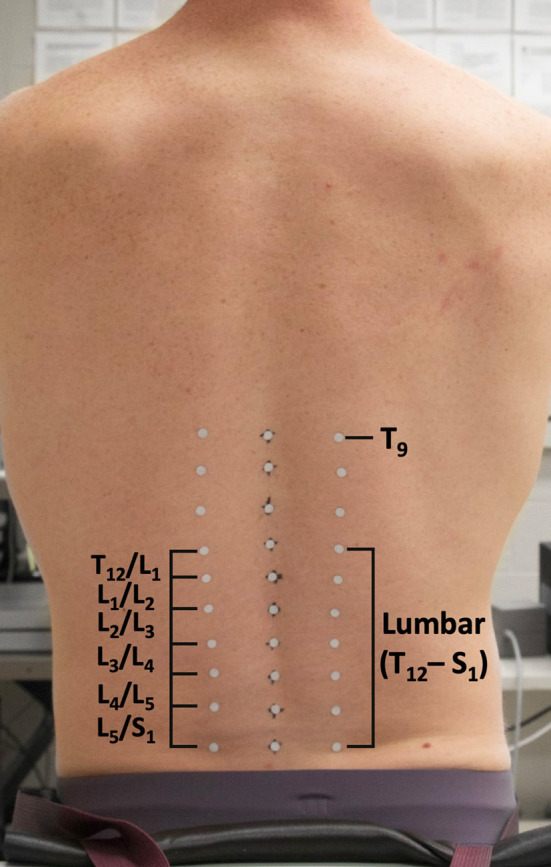
Pictorial depiction of reflective marker setup for the intersegmental method.

Participants were outfitted with three columns of 10 reflective markers (6 mm diameter) placed over the spine, with the middle column placed superficial to spinous processes from T_9_ to S_1_. Left and right columns were placed over the apex of the paraspinal muscles, 3–5 cm lateral to each spinous process marker (Figure 1). All kinematic data were collected at 120 Hz (Optitrack, NaturalPoint Inc. Corvalis, OR, USA).

### Dynamic Stability Protocol

**Figure 2:**
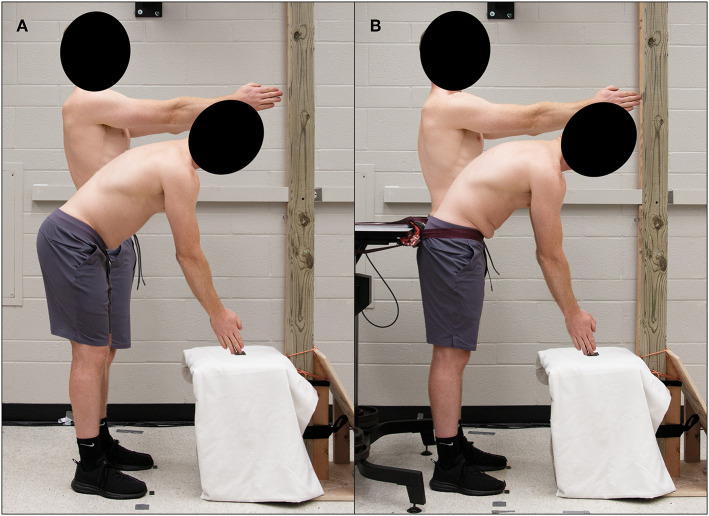
Experimental setup of Free Motion **(A)** and Pelvic Constrained **(B)** dynamic stability trials.

Participants completed two dynamic stability trials [Free Motion (FM), Pelvis Constrained (PC)] each consisting of 65 continuous cycles of repetitive spine flexion-extension movements at a rate of 0.25 Hz (Figures 2A,B, respectively). During each trial, participants were required to repeatedly touch two targets with their hands extended in front of them. Both targets were in the anterior sagittal midline of the body with the top target located at shoulder height and the bottom target located 50 cm anterior to the knee (Granata and England, 2006; Granata and Gottipati, 2008; Ross et al., 2015; Larson et al., 2018). During the PC trial, a belt was placed across the participants' anterior superior iliac spines to secure their pelvis to a rigid table and therefore isolate motion to the spine; the FM trial was not constrained in any way thereby allowing for lower limb and pelvic movement in conjunction with spine motion. The order of the two trials was randomized and counterbalanced across the study with 20 min of rest between trials. Immediately prior to and following each trial, participants were asked to rate their perceived exertion level (RPE) on a modified Borg scale (0–10).

### Data Processing and Analysis

Using a custom intersegmental model (Zwambag et al., 2018), a cubic polynomial spline, comprised of four knots and five segments, was fit to each column using the X, Y, and Z positions of reflective markers from T_9_ to S_1_. Local coordinate systems (LCS) were created at each spine level with the origin of each LCS being located over each marker along the middle column of the spine. Using a flexion-extension, lateral bend, axial twist Cardan rotation sequence relating superior vertebral markers to inferior vertebral markers, regional 3D lumbar (T_12_ relative to S_1_) and intersegmental spine angles (e.g., T_12_ relative to L_1_) from T_9_/T_10_ through to L_5_/S_1_ were calculated throughout the repetitive movements. Angular data were low-pass filtered (effective 4th order Butterworth) with a 10 Hz cut-off frequency (Granata and Gottipati, 2008; Larson et al., 2018). Angular motion at the T_9_T_10_, T_10_/T_11_, T_11_/T_12_ levels were smaller and less periodic in nature than the more caudal levels, therefore only T_12_/L_1_ to L_5_/S_1_ were analyzed further for the calculation of Lyapunov exponents (LyE).

**Figure 3:**
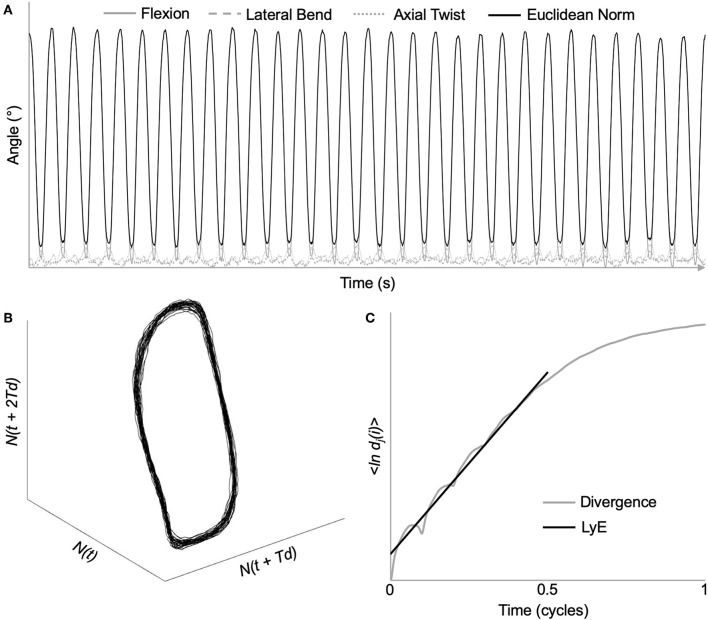
Demonstration of maximum Lyapunov exponent analysis using time series data from a representative L_2_/L_3_ intersegmental motion segment. **(A)** Original 3D kinematic data, as well as the Euclidean norm throughout the dynamic stability trial. **(B)** Reconstructed state space using the method of delays [embedding dimension = 6; time delay = 48 samples (10% of movement cycle)]. **(C)** Average logarithmic divergence of all nearest neighbors. Maximum Lyapunov exponents (LyE) calculated using the slope of the line fitted from 0 to 0.5 movement cycles.

To assess the neuromuscular control of lumbar and intersegmental spine movements, local dynamic stability of the spine was determined using the maximum finite-cycle LyE. The first five movement cycles were removed to ensure that steady-state dynamic movement was analyzed (Graham and Brown, 2012). As LyE may be affected by time series length (Bruijn et al., 2009b), 3D angular data from the next 30, 40, and 50 movement cycles were time normalized to 14,400, 19,200, 24,000 samples (120 Hz * 4 s/cycle * # of cycles), respectively. This ensures that cycle to cycle temporal variability is maintained as the number of movement cycles analyzed increases. Due to the elevated Borg RPE following the completion of each trial (shown in Results) as well as some participants describing a feeling of fatigue toward the end of the 65 cycles, angular data from the full 60 movement cycles were not analyzed to mitigate the potential effect of fatigue on LyE. Prior to calculating the Euclidean norm, otherwise known as the root-sum-square, all three spine angles were shifted into positive space to preserve the original angular displacement waveform characteristics (Beaudette et al., 2016) (Figure 3A). All these analyses were completed using custom MATLAB programs (The MathWorks, Natick, MA, USA).

Using the method of delays, the complex dynamical system represented by the Euclidean norm was time delay embedded to provide a reconstructed state space (Figure 3B) through the following equation:

$$\begin{array}{l} Y\left(t\right)=\left[N\left(t\right),\;N\left(t+Td\right),\;N\left(t+2Td\right),\ldots ,N\left(t+\left(n-1\right)Td\right)\right] \end{array}$$

where *Y*(*t*) is the n-dimensional state space, *N*(*t*) is the original Euclidean norm data, *T*_*d*_ is the constant time delay, and *n* is the number of reconstructed dimensions (Abarbanel et al., 1993).

Based on previous research, a six-dimensional state space reconstruction and a time delay of 10% of cycle (Granata and England, 2006; Graham et al., 2014; Ross et al., 2015) was chosen to ensure that all data were processed similarly. From the reconstructed state space, LyE were then calculated by analyzing the exponential rate of divergence of nearest neighboring trajectories using the following equation:

$$\begin{array}{l} y\left(i\right)=\frac{1}{\Delta t}\left\{\text{ln }dj\left(i\right)\right\} \end{array}$$

where {ln *d*_*j*_(*i*)} represents the average logarithmic divergence, *d*_*j*_(*i*), for all pairs of nearest neighbors, *j*, throughout a certain number of time delays, Δt (Rosenstein et al., 1993). The slope of the linear best fit line was calculated from 0 to 0.5 cycles (Bruijn et al., 2009a) (Figure 3C).

Additionally, maximum lumbar and intersegmental Euclidean norm ranges of motion (ROM) were determined for each individual cycle and averaged across each entire trial.

### Statistical Analysis

Initially, differences in lumbar and intersegmental LyE between the number of cycles analyzed (30, 40, 50) were determined using one-way repeated measures ANOVAs. As this revealed no significant difference among the number of cycles analyzed (shown in Results), all subsequent statistical tests included LyE from only 30 cycles. This aligns with previous studies (e.g., Dupeyron et al., 2013) and avoids any potential effects of accumulated fatigue (due to the higher number of cycles) on LyE. A two-way repeated measures ANOVA was used to determine if total lumbar (T_12_/S_1_) ROM and LyE were different between trials (PC, FM) and sexes (M, F). Last, a three-way repeated measures ANOVA was used to determine any differences between trial (PC, FM), sex (M, F), and intersegmental level (T_12_/L_1_, L_1_/L_2_, L_2_/L_3_, L_3_/L_4_, L_4_/L_5_, L_5_/S_1_) ROM and LyE. In all ANOVAs, participants were modeled as random effects. *Post-hoc* pairwise multiple means comparisons were applied using a Tukey adjustment where ANOVAs reported statistically significant main effects (α = 0.05). All statistical analyses were performed using SAS University Edition (SAS Institute, Cary, NC, USA).

## Results

### Methodological Considerations

**Figure 4:**
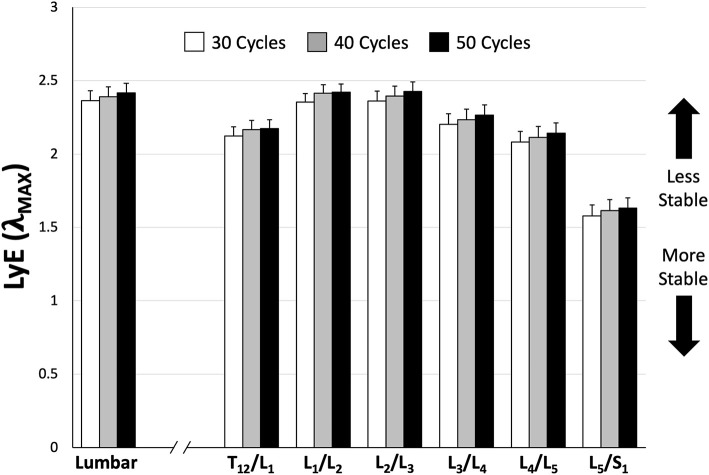
Mean (±SEM) lumbar and intersegmental maximum finite-cycle Lyapunov exponents (LyE) comparing the number of cycles included in the analysis. Note that lower LyE values represent greater stability.

There we no significant main or interaction effects detected when analyzing lumbar ROM. However, for intersegmental ROM there was a statistically significant trial*sex interaction effect [*F*_(1, 154)_ = 6.69, *p* = 0.0106]. Specifically, male intersegmental ROM was significantly greater during the PC compared to FM trial, while there were no significant differences between trials for females or between sexes within trials (Table 2). Additionally, ROM was statistically different between segments [*F*_(5, 154)_ = 91.31, *p* < 0.0001] with the greatest intersegmental motion observed at L_3_/L_4_ and L_4_/L_5_ segments across all trials (Table 2). Borg RPE ratings increased from 0.3 (±0.41) pre-trial to 4.0 (±2.09) post-trial and from 0.2 (±0.31) pre-trial to 3.9 (±2.24) post-trial following 65 cycles of the FM and PC trials, respectively. Across all participants, there was no significant difference in LyE across the number of cycles analyzed (30, 40, or 50) for both lumbar [*F*_(2, 78)_ = 0.59, *p* = 0.556] and intersegmental [*F*_(2, 558)_ = 1.62, *p* = 0.199] motion (Figure 4).

**Table 2:** Comparison of mean (±SEM) lumbar and intersegmental Euclidean norm range of motion (degrees) throughout 30 repetitive free motion or pelvis constrained flexion movements for both males and females.

**Segment**	**Free motion**		**Pelvis constrained**	
	**Female**	**Male**	**Female**	**Male**
Lumbar	42.9 (±2.01)	42.2 (±3.98)	41.2 (±4.82)	46.2 (±3.04)
T_12_/L_1_	5.2 (±0.49)^D^	4.2 (±0.50)^D^^*^	4.7 (±0.80)^D^	4.7 (±0.38)^D^^*^
L_1_/L_2_	6.4 (±0.34)^C^	5.7 (±0.45)^C^^*^	5.9 (±0.85)^C^	6.3 (±0.39)^C^^*^
L_2_/L_3_	8.0 (±0.36)^B^	7.6 (±0.60)^B^^*^	7.6 (±0.93)^B^	8.4 (±0.49)^B^^*^
L_3_/L_4_	9.9 (±0.56)^A^	10.1 (±0.75)^A^^*^	9.3 (±1.07)^A^	11.0 (±0.75)^A^^*^
L_4_/L_5_	8.6 (±0.63)^A^	9.4 (±1.06)^A^^*^	8.6 (±1.00)^A^	10.1 (±0.91)^A^^*^
L_5_/S_1_	4.3 (±0.39)^D^	4.4 (±0.58)^D^^*^	4.3 (±0.60)^D^	4.9 (±0.56)^D^^*^

*Different letters indicate statistically significant differences between segments for both males and females (p < 0.05) (e.g., T_12_/L_1_ and L_5_/S_1_ are not significantly different from each other and both are significantly different than all other motion segments)*.

* *Statically significant difference between trials with the same symbol (males only) (p < 0.05) (e.g., males demonstrated significantly greater range of motion during the pelvis constrained trial)*.

### Whole Lumbar Stability

**Figure 5:**
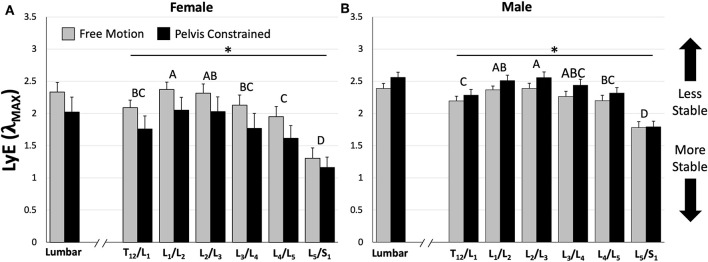
Comparison of mean (±SEM) lumbar and intersegmental maximum finite-cycle Lyapunov exponents (LyE) for females **(A)** and males **(B)** between dynamic stability trials. Note that lower LyE values indicate greater stability. The asterisk (*) above the horizontal lines represents a statistically significant difference between dynamic stability trials within sexes (e.g., females demonstrated greater stability during the pelvis constrained trial). Different capital letters above the bars indicate statistically significant differences between intersegmental levels within each sex (e.g., L_5_/S_1_ is significantly different than all other motion segments for both sexes). Also note that females demonstrated significantly greater stability during the pelvis constrained trial compared to males for both lumbar and intersegmental levels; however, their significance is not shown for clarity (*p* < 0.05).

Analysis of whole lumbar LyE from 30 repetitive flexion cycles revealed a significant trial*sex interaction effect [*F*_(1, 14)_ = 9.58, *p* = 0.0079]. Specifically, females were significantly more stable (lower LyE) than males during the PC trial (Figure 5); however, no significant differences between sexes were observed during the FM trial (Figure 5). Although not statistically significant, females demonstrated greater lumbar stability (lower LyE) during the PC trial (Figure 5A), while males demonstrated decreased lumbar stability (higher LyE) during PC compared to the FM trial (Figure 5B).

### Intersegmental Stability

Across all intersegmental levels, a significant trial*sex interaction [*F*_(1, 154)_ = 56.6, *p* < 0.0001] was observed, with females demonstrating significantly greater stability (lower LyE) then males during the PC trial and no significant differences during the FM trial (Figure 5). Additionally, females exhibited significantly greater stability (lower LyE) during the PC compared to the FM trial (Figure 5A), while males were significantly less stable (higher LyE) in the PC compared to the FM trial (Figure 5B). Last, a significant intersegmental level*sex interaction [*F*_(5, 154)_ = 3.22, *p* = 0.0085] was found. Despite this interaction, for both sexes, the L_5_/S_1_ motion segment exhibited the greatest stability (lower LyE) across both PC and FM trials (Figure 5).

## Discussion

The primary goal of the current study was to calculate and compare dynamic stability amongst lumbar spine sub-regions (i.e., intersegmentally); the data demonstrated that the most caudal region of the spine behaved the most dynamically stable across both movement conditions. The secondary goal of the study was to determine if a pelvic constraint affected different sub-regions of the lumbar spine similarly. This was confirmed; however, the pelvis constraint influenced male and female dynamic stability differently. Finally, the results demonstrated that 30 repetitive flexion-extension cycles produced both whole lumbar and intersegmental dynamic stability values that were not different from those obtained using higher numbers of cycles (Figure 4), which aligns with previous research (Dupeyron et al., 2013).

When considering intersegmental stability, it was hypothesized that lower lumbar vertebral segments (e.g., L_4_/L_5_) would demonstrate the lowest dynamic stability (higher LyE), due to lower back pain and injury often being linked to abnormal intervertebral spine motion/stability (Cholewicki and McGill, 1996; McGill and Cholewicki, 2001) and greater instability and degeneration being observed at lower vertebral levels (Friberg and Hirsch, 1949). However, in actuality the upper lumbar motion segments (e.g., L_1_/L_2_ and L_2_/L_3_) demonstrated the lowest dynamic stability (higher LyE) while the most caudal (L_5_/S_1_) motion segment demonstrated the greatest dynamic stability (lower LyE) across both sexes (Figure 5). These observations were not dependent on whether or not the pelvis was constrained. Similarly, a previous study demonstrated that the most caudal spine regions displayed more deterministic kinematics (using recurrence quantification) than the more cranial regions (Dideriksen et al., 2014). It is interesting to consider the skeletal anatomy in light of this finding of the current study. The lower thoracic and lumbar vertebrae span the distance between the thoracic cage and sacrum (i.e., rigid skeletal structures) with the upper lumbar motion segments (L_1_/L_2_ and L_2_/L_3_) located approximately in the middle (as the floating ribs would not be considered rigid). Due to their location away from these rigid skeletal structures, these motion segments may be subject to a greater potential for kinematic disturbances due to the greater number of degrees of freedom immediately influencing the segment, thereby resulting in lower dynamic stability (higher LyE). As the distance of the motion segments to these rigid skeletal structures decreases, the number of nearby degrees of freedom and the potential for kinematic disturbances may also decrease, which may ultimately result in easier control and greater dynamic stability (lower LyE). It must be noted that local dynamic stability, which provides information about the behavior of kinematic variance, is fundamentally different than mechanical stability which describes the potential for mechanical buckling. As lower lumbar intervertebral levels generally experience higher compressive (and destabilizing) forces, the likelihood of buckling and mechanically unstable behavior may still be highest in the lower lumbar levels. Regardless, it is interesting that the upper lumbar regions display less stable control behavior during these flexion-extension motions; the implications of this need to be further studied.

With regard to the effect of the pelvis constraint, our hypothesis that dynamic stability would be greater when the pelvis was constrained was not completely supported. Across our sample population (n = 16), there were no significant differences in LyE between FM and PC trials; however, a significant trial by sex interaction revealed a more complex relationship. More specifically, females (n = 6) demonstrated significantly greater intersegmental stability (lower LyE) in the PC trial compared to FM trial (Figure 5A), while males (n = 10) demonstrated a relatively small but statistically significant decrease in intersegmental stability when the pelvis was constrained (Figure 5B). This sex-based interaction has not been reported by previous studies that have compared FM and PC conditions on dynamic spine stability (Granata and Gottipati, 2008; Howarth and Graham, 2015). Specifically, Granata and Gottipati (2008) reported a significant decrease in dynamic stability (higher LyE) when the pelvis was constrained as well as no significant main effect of sex, but did not appear to test for an interaction between them, while Howarth and Graham (2015) reported a significant increase in dynamic stability (lower LyE) when the pelvis was constrained but did not test for an effect of sex. Another novel finding of the current study is that the pelvic constraint did not have a differential effect on any specific intersegmental level. Because the pelvic constraint was located in closest proximity to the lower lumbar segments, it was thought that it might have a larger effect on these specific levels. However, the results demonstrated that this was not the case. Although the pelvic constraint has been described to “simplify” motion and effectively isolate movement to the spine, only males demonstrated statistically significant effects of the pelvic constraint on intersegmental spine ROM during the repetitive flexion movements (Table 2). During the constrained condition, mean intersegmental ROM increased by 0.63 degrees (8.9% change) in males, while females decreased ROM by a non-statistically significant mean of 0.33 degrees (5.4% change). Whole lumbar spine ROM differences in this constrained condition were small [male mean increase of 4 degrees (8.6% change) and female mean decrease of 1.7 degrees (4.0% change)] and not statistically significant, indicating that spine motion was similar in both movement conditions as the magnitude was not significantly influenced by the pelvic constraint. Previous research has also demonstrated that ROM magnitude does not inherently influence LyE (Gsell et al., 2015). However, co-variates often associated with sex differences, including height and weight, could influence this effect as our female sample population were considerably shorter and lighter than the male participants. To assess the likelihood that these co-variates may have influenced the results of the current study, we divided our male sample population into groups based on height and mass (five tallest and five shortest; five heaviest and five lightest) and compared LyE within each. Both tall and short groups, as well as heavy and light groups, decreased (higher LyE) (4.3% 10.7% for tall and short; 8.3% and 6.4% for heavy and light) dynamic lumbar spine stability when the pelvis was constrained. This suggests that the observed sex differences were more likely due to differing control strategies, system dynamics, and/or anatomical differences beyond height and weight. Future work is needed to study this further.

When interpreting the results of this study, there are some limitations to consider. The first limitation concerns the use of skin mounted kinematic tracking during dynamic movements. Although the kinematic model used in the current study has been shown to be robust to marker noise during spine flexion (Zwambag et al., 2018), the vertebral positions and motion can only be approximated using skin surface markers. Thus, while we have labeled lumbar segments based on their position in standing (i.e., T_12_/L_1_ to L_5_/S_1_), the skin mounted markers would not perfectly track these specific segments throughout the full range of motion. Therefore, while we can confidently say for example that the most caudal lumbar segment behaved in the most dynamically stable manner, we cannot say that this definitively represents the L_5_/S_1_ intervertebral motion segment. A second limitation involves methodological considerations surrounding the parameters involved in state space reconstruction when calculating LyE. In the current study, six embedding dimensions and a time delay of 10% were used for all Euclidean norm kinematic data as these have been used in many previous spine dynamic stability studies (e.g., Granata and England, 2006; Graham et al., 2014; Ross et al., 2015); however, these set parameters may not best represent the underlying structure of each complex signal or system being analyzed. As previous studies have eluded (e.g., Granata and Gottipati, 2008; Graham et al., 2011; Asgari et al., 2015), some signals may contain greater dynamic complexity and therefore need to be reconstructed with parameters of differing values. To test this for our data set, time delays and embedding dimensions were additionally calculated using the mutual average information function (Fraser and Swinney, 1986) and global false nearest neighbor analysis (Kennel et al., 1992), respectively, followed by a time delay recalculation for the given embedding dimension using the average displacement technique described by Rosenstein et al. (1994). This was completed for whole lumbar and intersegmental motions and revealed that both embedding dimensions and time delays showed some variation amongst participants, trials, and motion segments (Table 3); however, the means were not substantially different than the values of 6 dimensions and 10% delay used in the main analysis. Future studies should continue to investigate the use of individual state space reconstruction parameters on LyE to ensure that all data are properly characterized and represented for dynamic stability analysis.

**Table 3:** Comparison of mean (±SD) lumbar and intersegmental embedding dimensions (ED) and time delay (T_d_) calculated for 30 repetitive free motion or pelvis constrained flexion movements.

**Segment**	**Free motion**		**Pelvis constrained**	
	**ED**	**T_**d**_ (%)**	**ED**	**T_**d**_ (%)**
Lumbar	6.1 (±0.85)	10.2 (±1.53)	6.6 (±0.81)	9.3 (±1.20)
T12/L1	6.1 (±0.89)	10.1 (±1.41)	6.4 (±0.96)	9.7 (±1.53)
L1/L2	5.9 (±0.72)	10.5 (±1.43)	6.6 (±1.09)	9.4 (±1.53)
L2/L3	6.0 (±0.82)	10.3 (±1.39)	6.4 (±0.96)	9.5 (±1.41)
L3/L4	6.2 (±0.98)	10.0 (±1.59)	6.5 (±1.10)	9.5 (±1.87)
L4/L5	6.4 (±0.88)	9.6 (±1.48)	6.8 (±0.91)	8.9 (±1.13)
L5/S1	6.3 (±0.48)	9.3 (±0.84)	6.7 (±0.87)	8.6 (±1.15)

In conclusion, results demonstrate that the most caudal region of the lumbar spine behaves as the most dynamically stable while upper lumbar regions behave the most dynamically unstable during repeated standing trunk flexion-extension movements. These findings may be related to each region's proximity to rigid skeletal structures (i.e., thoracic cage and sacrum); however, more studies are needed to probe this further. Further, constraining pelvic motion significantly affects male and female whole lumbar and intersegmental dynamic stability differently, with females demonstrating greater stability and males demonstrating decreased stability compared to free motion. Therefore, consideration is needed when interpreting motion of the lumbar spine, as a pelvic constraint may effectively function to isolate more motion to the spine, but this may be less representative of normal spine motion during everyday tasks.

## Data Availability Statement

The datasets generated for this study are available on request to the corresponding author.

## Ethics Statement

The studies involving human participants were reviewed and approved by University of Guelph Research Ethics Board. The patients/participants provided their written informed consent to participate in this study.

## Author Contributions

DL and SB contributed to conception and design of the experiment. DL, YW, and DZ performed data collection and analysis. DL and SB contributed to drafting the manuscript. All authors contributed to manuscript revision and approved the submitted version.

### Conflict of Interest

The authors declare that the research was conducted in the absence of any commercial or financial relationships that could be construed as a potential conflict of interest.
